# Traumatic rupture of a hepatic hydatid cyst in a polytrauma patient: a case report highlighting diagnostic and management challenges

**DOI:** 10.1097/RC9.0000000000000538

**Published:** 2026-05-26

**Authors:** Nur Alkrinawi, Sofyan Abu Freih, Guy Barsky, Anton Osyntsov, Gadi Shaked

**Affiliations:** a Department of General Surgery B, Soroka University Medical Center, Be’er Sheva, Israel; b Medical School For International Health, Ben-Gurion University of the Negev, Be’er Sheva, Israel

**Keywords:** echinococcosis, hydatid disease, liver cyst rupture, polytrauma, trauma

## Abstract

**Introduction and importance:**

Traumatic rupture of hepatic hydatid cysts is a rare but life-threatening complication of echinococcal disease. Rupture following blunt abdominal trauma can lead to anaphylaxis, massive peritoneal contamination, and rapid hemodynamic deterioration. Early recognition is essential, especially in endemic regions.

**Case presentation:**

A 19-year-old, previously healthy male sustained polytrauma in a high-speed motor vehicle accident. Initial assessment showed a Glasgow Coma Scale of 3 and a fixed left pupil. Computed tomography demonstrated a left subdural hemorrhage and bilateral subarachnoid hemorrhages (Fig. 1A), a traumatic rupture of a hepatic hydatid cyst with peritoneal spillage (Fig. 1B), a suspected jejunal injury, hemoperitoneum, and an L4 vertebral fracture (Fig. 1C). The patient underwent emergent exploratory laparotomy with evacuation of daughter cysts, cyst unroofing, hypertonic saline lavage, and repair of a jejunal laceration, as well as decompressive craniectomy. Despite aggressive resuscitation and critical care, the patient developed refractory intracranial hypertension and died on postoperative day two, primarily due to severe traumatic brain injury, with hydatid cyst rupture contributing to the overall clinical complexity.

**Figure 1.**
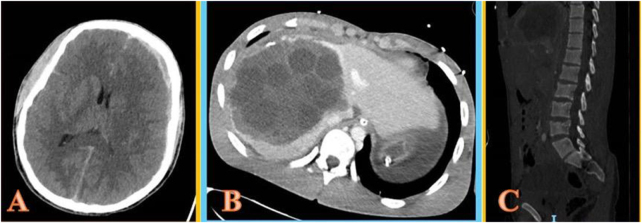
Whole-body contrast CT. (A) Left subdural and bilateral subarachnoid hemorrhages with midline shift. (B) Traumatic rupture of a hepatic echinococcal cyst with peritoneal dissemination and active bleeding. (C) L4 vertebral fracture.

**Clinical discussion:**

Traumatic rupture of hepatic hydatid cysts is uncommon but is associated with significant morbidity and mortality. Diagnosis may be delayed in polytrauma patients due to competing injuries that obscure clinical signs. Peritoneal dissemination may result in anaphylaxis, sepsis, and recurrence. Even with timely surgical intervention, outcomes are often determined by associated life-threatening injuries, such as severe traumatic brain injury, as in this case.

**Conclusion:**

Traumatic rupture of hepatic hydatid cysts should be considered in patients with blunt abdominal trauma in endemic regions. Early imaging, prompt surgical intervention, and a high index of suspicion are crucial. In polytrauma patients, this significantly complicates clinical prioritization and may determine prognosis, even when abdominal contamination is surgically controlled.

## Introduction

Hydatid disease is a zoonotic disease caused by the Echinococcus parasite, which is a member of the Taeniidae family. The most common Echinococcus species that cause hydatid disease in humans are *Echinococcus granulosus* (cystic echinococcosis) and *Echinococcus multilocularis* (alveolar echinococcosis)^[^1^]^.

The World Health Organization (WHO) estimates that more than one million people are affected by echinococcosis worldwide, and the disease remains endemic in regions such as the Middle East, China, Central Asia, Eastern Europe, and South America, where livestock serve as intermediate hosts and canines as definitive hosts^[^2^]^.

HIGHLIGHTS

- Traumatic rupture of hepatic hydatid cysts is rare and often lethal in endemic areas.
- Polytrauma may obscure the diagnosis, especially in the absence of a previously known hepatic cyst.
- Early surgical intervention with evacuation and peritoneal lavage is essential after intraperitoneal spillage.
- Hemodynamic instability may be multifactorial, including hemorrhagic, septic, and anaphylactic mechanisms.
- Severe associated injuries, particularly traumatic brain injury, may determine the overall prognosis.

Humans become accidental intermediate hosts through the ingestion of parasite eggs via contaminated food or direct contact with infected canids. Following ingestion, the eggs hatch in the intestine, releasing oncospheres that penetrate the intestinal wall and migrate via the portal or lymphatic circulation to various organs, where they develop into hydatid cysts^[^3,4^]^.

Hydatid disease can develop in almost all organs and tissues of the human body, but the most frequently involved organs are the liver (50%-77%), lungs (15%-47%), spleen (0.5%-8%), and kidneys (2%-4%). Most hepatic hydatid cysts remain asymptomatic for years and are often detected incidentally; however, symptoms may develop as cysts enlarge or complications occur^[^5,6^]^.

The major complications of hepatic hydatid cysts are rupture and secondary bacterial infection. Primary peritoneal hydatidosis is rare (2%), and the mechanism of this infection is unknown.

Intraperitoneal rupture is uncommon but life-threatening, occurring either spontaneously due to increased intracystic pressure or following trauma. Superficially located cysts, large cysts, and viable cysts with high pressure are especially prone to rupture into body cavities such as the pleural space or peritoneal cavity, or may drain into the biliary tract or into the gastrointestinal system.

Traumatic rupture into these spaces is associated with severe consequences, including anaphylactic shock, secondary bacterial infection, and dissemination of the parasite^[^7–9^]^.

The main diagnostic methods are ultrasonography (US) and computed tomography (CT). Presentation is usually dramatic, with acute abdominal signs such as guarding, rebound, and tenderness generally present. This complication should be included in the differential diagnosis of acute abdomen, especially in endemic areas.

The modern treatment of hydatid cysts of the liver varies from surgical intervention to percutaneous drainage or medical therapy. Surgery is still the treatment of choice and can be performed using either the conventional or laparoscopic approach. Percutaneous drainage and treatment of the cyst with hypertonic saline or alcohol seem to be good alternatives to surgery in selected cases (PAIR: puncture, aspiration, injection of scolicidal agent, and reaspiration). Medical therapy with albendazole is typically initiated before and continued after invasive procedures. Intraperitoneal rupture is a life-threatening complication of hydatid cysts. It is important to manage patients with surgical intervention as soon as possible, along with aggressive medical treatment for anaphylactic reactions^[^9,10^]^.

Here, we present a fatal case of traumatic rupture of a hepatic hydatid cyst following blunt abdominal trauma in a young male with polytrauma injuries, highlighting the complexity of clinical prioritization in the presence of competing life-threatening conditions.

This case report has been reported in line with the SCARE 2025 criteria^[^11^]^.

## Case presentation

### Initial assessment

A 19-year-old Israeli male with no known medical history, including no prior history of hepatic hydatid disease or previously identified liver cysts, was brought to the Emergency Department 10 minutes after a high-speed motor vehicle accident. Upon arrival, he was intubated and sedated, with a Glasgow Coma Scale score of 3. He was hemodynamically stable (heart rate: 56 bpm, blood pressure: 119/53 mmHg), with a left-sided fixed dilated pupil. A seatbelt mark was noted across the abdomen; the abdominal examination revealed a soft but distended abdomen with no external bleeding.

Initial trauma room management included two large-bore intravenous lines, fluid resuscitation, and blood sampling. Focused Assessment with Sonography for Trauma (FAST) was positive for intra-abdominal fluid.

### Imaging findings

A full-body computed tomography (CT) scan revealed:

- Left subdural hemorrhage and bilateral subarachnoid hemorrhages, with midline shift (Fig. 1a)
- Traumatic rupture of a hepatic echinococcal cyst with peritoneal dissemination and active bleeding (Fig. 1B)
- L4 vertebral fracture (Fig. 1C)
- Suspected jejunal loop injury
- Large-volume hemoperitoneum

### Intraoperative findings

Following imaging, the patient was taken emergently to the operating room. At anesthesia induction, he became hypotensive and tachycardic; hemoglobin at that point was 14 g/dL. A fluid challenge and vasopressor support with adrenaline were initiated. Concurrent exploratory laparotomy and neurosurgical intervention (craniectomy) were performed.

All procedures were performed by senior trauma and neurosurgical teams at our Level 1 trauma center.

**Figure 2.**
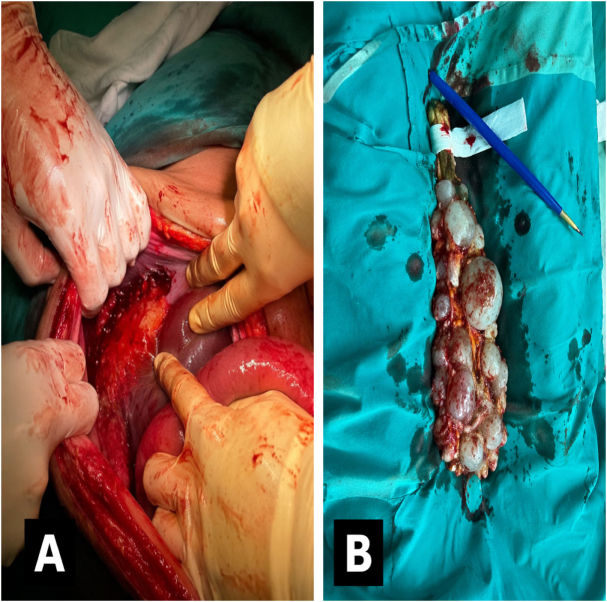
Intraoperative findings. (A) Ruptured hepatic hydatid cyst. (B) Evacuated peritoneal daughter cysts.

Intraoperative findings included:

- Hemoperitoneum of approximately 400 mL
- Ruptured hepatic hydatid cyst (Fig. 2A)
- Peritoneal spillage of daughter cysts (Fig. 2B)
- Retroperitoneal hematoma (Zone II, left)
- Jejunal laceration without mesenteric involvement

Surgical procedures included removal of daughter cysts from the abdominal cavity, unroofing of the liver cyst (Fig. 2), peritoneal lavage two times with 3% hypertonic saline for 15 minutes, and repair of the jejunal laceration. Two intra-abdominal drains were placed. Simultaneously, a fronto-parietal craniotomy and evacuation of the subdural hematoma were performed, followed by the insertion of an intracranial pressure (ICP) monitor. ICP readings remained critically high (60–80 mmHg). Pupils remained non-reactive postoperatively.

### Postoperative course

Postoperatively, the patient was admitted to the ICU on vasopressor support. Despite aggressive management, no neurological or hemodynamic improvement was observed. A repeat head CT on day two confirmed severe diffuse cerebral edema. The patient was declared dead later that day after cardiac arrest with absence of electrical and mechanical cardiac activity. Due to the patient’s early postoperative death, long-term follow-up and assessment of recurrence could not be performed.

### A timeline of key clinical events is summarized below:

Time 0: high-speed motor vehicle accident

- +10 minutes: arrival to Emergency Department; initial assessment: GCS 3, left-sided fixed, dilated pupil, FAST positive.
- + ~ 30 minutes: whole-body CT was performed.
- + ~ 60 minutes: transfer to operating room: Exploratory laparotomy and decompressive craniectomy.
- POD 2: death due to diffuse cerebral edema secondary to traumatic brain injury.

## Discussion

Traumatic rupture of hepatic hydatid cysts is rare, with only a limited number of cases reported in the literature. Most cases describe isolated abdominal presentations; however, rupture occurring in the setting of severe polytrauma is exceptionally uncommon and presents significant diagnostic and therapeutic challenges^[^1,9^]^.

Unlike most previously reported cases, which predominantly describe isolated abdominal involvement, this case demonstrates hydatid cyst rupture in the context of severe polytrauma, where competing life-threatening injuries complicate both diagnosis and management. This highlights the challenge of clinical prioritization and the need for rapid multidisciplinary decision-making.

Intraperitoneal hydatid cyst rupture may occur following trauma or spontaneously due to increased intracystic pressure, and rarely, it may be iatrogenic during elective surgery^[^1,6^]^. Some studies have concluded that most intraperitoneal ruptures develop after trauma; nevertheless, other studies have pointed out that spontaneous ruptures may develop more frequently^[^12–14^]^.

Risk factors for rupture include young age, large cyst size (>10 cm), superficial liver location, and trauma^[^1,15,16^]^.

Similar to previously reported cases, the patient presented with peritoneal dissemination and hemodynamic instability. However, the coexistence of severe traumatic brain injury created a unique clinical challenge, where prioritization of life-threatening conditions became critical. Moreover, the absence of a previously known hepatic cyst further complicated the diagnostic process, particularly in the setting of severe polytrauma, where competing injuries may obscure the underlying pathology.

The screening tool for hydatid cysts is mainly the US. In cases of hydatid cyst perforation, US shows intra-abdominal fluid. It might also demonstrate intraperitoneal cysts. Nevertheless, CT plays a key role in urgent or trauma settings and allows for multiorgan screening. Both modalities should be able to display heterogeneous cavities or cystic structures in the liver^[^5,9,16^]^.

In trauma patients presenting with hemoperitoneum, a broad differential diagnosis should be considered, especially in endemic regions. Possible causes include hepatic laceration, splenic injury, mesenteric injury, bowel perforation, and rupture of hepatic cystic lesions, including hydatid cysts.

Hemodynamic instability in such cases is often multifactorial and may result from hemorrhagic shock, septic shock, or a possible anaphylactic reaction secondary to hydatid fluid spillage. In the present case, the relatively small volume of hemoperitoneum supports a multifactorial etiology, with a suspected anaphylactic component.

Peritoneal dissemination following rupture may lead to anaphylaxis, peritonitis, and secondary echinococcosis^[^17^]^. The primary aims of emergent surgical treatment are to control contamination, minimize the risk of anaphylactic reactions, and prevent the development of secondary peritoneal hydatidosis^[^16,18^]^.

Medical treatment for intraperitoneal rupture should be initiated as soon as possible at the emergency unit after confirming the diagnosis of intraperitoneal rupture. Moreover, it should be continued during surgery. To minimize morbidity and mortality, patients should be hemodynamically stabilized before surgery, and they should undergo surgery as soon as possible. Medical treatment is aimed at stabilizing hemodynamic status with fluid resuscitation and treating anaphylactic reactions with corticosteroids, antihistamines, and vasopressor drugs^[^1,13,14^]^.

There is no consensus in the literature about surgical treatment options for intraperitoneal cyst rupture. Although international guidelines, such as those from the WHO and the Centers for Disease Control, provide recommendations for the general management of cystic echinococcosis, they do not specifically address traumatic or peritoneal rupture^[^19,20^]^.

We suggest that each case should be evaluated separately, in accordance with the general principles of hydatid cyst surgery and the severity of associated traumatic injuries.

Cyst contents that trigger anaphylactic reactions should be removed from the abdominal cavity as soon as possible. The peritoneal cavity should be washed with scolicidal solutions such as formaldehyde, hypertonic saline (3%–10%–15%-30%), silver nitrate (0.5%), cetrimide, chlorhexidine, cetrimide plus chlorhexidine (1.5%/0.15%), hydrogen peroxide (1.5%–3%), povidone iodine (10%–50%), or ethyl alcohol (70%–95%). Each solution has a different time frame for possible scolicidal effects^[^1,6,13,18^]^.

We prefer hypertonic saline to wash the peritoneal cavity at least two times, with each wash lasting 10–15 minutes. Allergens that lead to anaphylactic reactions can be removed in this manner.

Perforated cystic cavities should be carefully evaluated. Remaining cystic contents should also be evacuated, and the free edges of the cystic cavity should be widely excised. Abdominal drains should be placed both into the cystic cavity and in the abdomen before the completion of surgery.

Postoperatively, antihelminthic treatment with albendazole (10–15 mg/kg/day) should be administered as soon as possible for patients diagnosed with intraperitoneal rupture to prevent disease recurrence due to overlooked cystic contents during surgery. Clinical follow-up should be performed regularly, particularly during the early postoperative period, and continued annually in the long term when feasible^[^1,13^]^.

In the present case, the coexistence of severe traumatic brain injury significantly influenced surgical decision-making and postoperative management, limiting the ability to initiate adjunctive antiparasitic therapy and ultimately contributing to the fatal outcome.

Recurrence after intraperitoneal rupture of hepatic hydatid cysts has been reported to range from 0% to 28%, reflecting the risk of peritoneal dissemination and incomplete surgical clearance.

Mortality has been reported in patients with ruptured hepatic hydatid cysts, particularly in small case series and in cases complicated by anaphylaxis or delayed diagnosis. However, exact mortality rates remain unclear due to the rarity of the condition.

In polytrauma settings, outcomes are primarily determined by associated life-threatening injuries^[^1,5,9,13^]^.

The patient’s rapid clinical deterioration and the presence of disseminated cyst contents prompted emergent surgical exploration. Although the abdominal source of contamination and a possible anaphylactic component were addressed, the fatal outcome was primarily attributable to severe concomitant traumatic brain injury.

In emergency trauma scenarios, management must be individualized according to the patient’s hemodynamic status and associated injuries. Damage control principles often take precedence, favoring rapid interventions aimed at stabilization rather than definitive surgical treatment.

This case adds to the limited body of literature by illustrating how a rare abdominal pathology can coexist with severe polytrauma, where clinical outcomes are determined not by a single condition but by the interaction of multiple life-threatening injuries. It reinforces the importance of prioritization, rapid decision-making, and coordinated multidisciplinary management in such complex scenarios.

### Limitations

This case highlights a rare presentation of traumatic hydatid cyst rupture in the setting of severe polytrauma, emphasizing diagnostic and therapeutic challenges. However, it is limited by its single-case nature and the inability to assess long-term outcomes due to the fatal course.

### Learning points

- Traumatic rupture of hepatic hydatid cysts should be considered in patients presenting with blunt abdominal trauma in endemic regions.
- Early recognition of intraperitoneal spillage is essential to enable prompt surgical intervention, including the evacuation of cyst contents and peritoneal lavage.
- In polytrauma patients, concomitant injuries may obscure the diagnosis, particularly in the absence of a previously known hepatic cyst.
- Hemodynamic instability in such cases is often multifactorial, with contributions from hemorrhagic, septic, and anaphylactic mechanisms.
- A high index of suspicion and coordinated multidisciplinary management are crucial to optimize outcomes.

## Conclusion

Traumatic rupture of hepatic hydatid cysts is a rare but potentially life-threatening complication, particularly in the setting of severe polytrauma. Clinicians in endemic regions should maintain a high index of suspicion in trauma patients presenting with hemoperitoneum or unexplained hemodynamic instability.

Early imaging and prompt surgical intervention are essential to control contamination, while hemodynamic instability may be multifactorial, including a possible anaphylactic component. In such complex cases, management should be individualized, with damage control principles and multidisciplinary coordination guiding decision-making.

## Data Availability

Data are not publicly available due to patient confidentiality but are available from the corresponding author upon reasonable request.

## Patient perspective

Due to the patient’s critical condition and subsequent death, a patient’s perspective could not be obtained.
